# Studying How Calcium Silicate and Radiopacifier Proportions Affect the Physicochemical Properties of Endodontic Calcium Silicate-Based Sealers

**DOI:** 10.3390/ma18184340

**Published:** 2025-09-17

**Authors:** Raimundo Sales de Oliveira Neto, Guilherme Ferreira da Silva, Tany Carvalho Moreira da Veiga, Stefani Jovedi Rosa, Brenda Stefhany Wilchenski de Souza, Rodrigo Ricci Vivan, Murilo Priori Alcalde, Marco Antonio Hungaro Duarte

**Affiliations:** Department of Operative Dentistry, Endodontics, and Dental Materials, Bauru School of Dentistry, University of São Paulo—USP, Bauru 17012-901, Brazil; guilherme.silva@fob.usp.br (G.F.d.S.); tany.fob022@usp.br (T.C.M.d.V.); stefanijovedi@usp.br (S.J.R.); brendawilchenski@usp.br (B.S.W.d.S.); rodrigo.vivan@fob.usp.br (R.R.V.); malcalde@fob.usp.br (M.P.A.); mhungaro@fob.usp.br (M.A.H.D.)

**Keywords:** calcium silicate, dental materials, physicochemical analysis, root canal obturation

## Abstract

This study evaluated how varying calcium silicate (30–50%) and radiopacifier (45–65%) ratios affected the physicochemical properties of experimental sealers (P1–P3) compared to Bio-C Sealer, AH Plus Bioceramic, and AH Plus Jet. Properties such as flowability, solubility, and radiopacity were assessed per ISO 6876/2012, and setting time followed ASTM C266-2008. pH and volumetric changes were measured in acrylic teeth (*n* = 10) filled with each sealer, with pH evaluated at 3, 24, 72, and 168 h, and volume changes assessed via micro-CT at baseline and after 7 days. P1 showed setting time similar to AH Plus Bioceramic (*p* > 0.05). All calcium silicate-based sealers had greater solubility than AH Plus Jet (*p* < 0.05), with only P3 meeting ISO standards. P1 and AH Plus Jet exhibited higher radiopacity (*p* < 0.05). Bio-C Sealer and AH Plus Bioceramic had more volume change than P2 (*p* < 0.05). All experimental sealers showed stable volume and increasing alkalinity, peaking at 72h. Bio-C Sealer showed a consistent pH rise, except between 24 and 168 h (*p* > 0.05). Sealer properties were strongly influenced by the composition. P1 (30% calcium silicate/65% radiopacifier) had superior flowability and radiopacity, while P3 (50% calcium silicate/45% radiopacifier) was the only one to meet solubility standards.

## 1. Introduction

The complete filling of the root canal system remains a major challenge in endodontics. Although various materials and techniques have been proposed, none have achieved this goal [1]. In the field of endodontic filling materials, calcium silicate-based sealers have gained considerable prominence due to their positive influence on the repair of periapical tissues, being recognized as bioactive materials [2,3]. The bioactivity is primarily attributed to their ability to release ions, particularly calcium and hydroxyl ions [4,5,6].

Calcium silicate-based sealers or hydraulic sealers are well recognized for their ability to induce periapical repair [7,8]. These materials offer long-lasting marginal sealing, high radiopacity, low setting expansion, significant ion-releasing capacity, and adequate working time for endodontic procedures [9,10,11]. Their biological properties are due to the formation of calcium hydroxide during the setting reaction and its subsequent dissociation [12]. However, these endodontic sealers have certain limitations, particularly regarding their manipulation and insertion into the root canal system, which stem from their grainy texture [1]. To overcome these drawbacks, incorporating propylene glycol and barium sulfate can improve plasticity, thereby enhancing its performance as a sealing material. These modifications can occur in the liquid (originally distilled water) and the powder, which initially consisted solely of Portland cement and bismuth oxide [1,13].

Another disadvantage of early calcium silicate-based sealers was the discoloration of the tooth structure. Collagen in the dentin matrix reacts with bismuth oxide, resulting in a grayish discoloration [14]. To address this issue, alternative radiopacifiers, such as tantalum oxide and zirconium oxide, have been suggested for use in endodontic cement. Studies have reported favorable results regarding tooth color stability when these radiopacifiers are used [15,16].

The AH Plus Bioceramic (Dentsply De Trey, Konstanz, Germany) is a premixed tricalcium silicate cement-based sealer that demonstrates adequate properties to be considered a good sealer but still has high solubility [17]. To date, the volumetric change in AH Plus Bioceramic has not been evaluated. Manufactured by the same company, AH Plus Jet (Dentsply De Trey, Konstanz, Germany) is an epoxy resin-based endodontic sealer marketed in a dual-syringe auto-mix system. It is commonly used as a benchmark for evaluating new sealers due to its excellent physical properties (such as dimensional stability, sealing ability, and flow) and its favorable chemical and biological characteristics, making it the gold standard in clinical practice [18].

Bio-C Sealer (Angelus, Londrina, Brazil) is a premixed hydraulic sealer specifically developed for root canal obturation. This ready-to-use material, supplied in a single syringe, contains calcium silicates, calcium aluminate, calcium oxide, zirconium oxide, iron oxide, silicon dioxide, and dispersing agents. Recent studies have demonstrated its superior cytocompatibility compared to AH Plus, showing enhanced cell viability, migration, morphology, adhesion, and mineralization capacity [19]. While exhibiting favorable physicochemical properties, including a short setting time, alkalinizing ability, adequate flow, satisfactory radiopacity, and minimal volumetric change, the sealer shows higher solubility than permitted by ISO 6876 standards [20].

Variations in the type and proportion of radiopacifiers can affect the physical and chemical properties of sealers [21]. Also, changes in the calcium content can contribute to the formation of calcium carbonates, which are essential for the bioactivity and biocompatibility of these materials [1,19,22]. Therefore, determining the optimal ratio of components in hydraulic endodontic sealer is crucial. This study evaluated the physical and chemical properties of calcium silicate-based sealers with varying proportions of calcium silicate and radiopacifiers (P1–P3), including setting time, radiopacity, flowability, solubility, volumetric change, and pH. They were compared with two commercially available sealers: AH Plus Jet and AH Plus Bioceramic. The null hypothesis was that there are no significant differences in the physicochemical properties between the P1, P2, and P3, and the commercial sealers AH Plus Jet and AH Plus Bioceramic, and that the different proportions of calcium silicate and radiopacifiers do not significantly affect these physicochemical properties.

## 2. Materials and Methods

The present study evaluated the physicochemical properties of calcium silicate-based sealers with varying proportions of calcium silicate and radiopacifiers (P1–P3). The composition of the tested sealers is detailed in Table 1. They were mixed to a consistency of obturation endodontic sealer, with a powder-to-liquid ratio of 2:1. AH Plus Bioceramic and Bio-C Sealaer were handled according to the manufacturer’s instructions. In contrast, AH Plus Jet was manually mixed by blending equal lengths (1 cm) of pastes A and B with a metal spatula on a mixing pad for 30 s, until a homogeneous consistency was achieved [23].

### 2.1. Setting Time

Initial and final setting times were determined according to ASTM C 266-2008 [24]. The experiment was conducted under controlled conditions of temperature (37 °C ± 1 °C) and humidity (95% ± 5%). Fifteen type IV gypsum rings, with an inner diameter of 10 mm and a thickness of 2.0 mm, were used to prepare the specimens (*n* = 3). The cements were placed in the rings that had been pre-soaked in distilled water. After 180 ± 5 s of mixing, the specimens were tested for setting by applying vertical pressure using a Gilmore needle, first with a 113.5 g needle for the initial setting and then with a 456.5 g needle for the final setting. The time elapsed from insertion into the gypsum rings to when no indentation was visible from either needle on the specimen surface was recorded.

### 2.2. Flowability

The flowability test was performed according to ISO 6876/2012 [25]. After mixing, 0.05 ± 0.005 mL of each cement was dispensed onto a glass plate using a disposable syringe. After 180 ± 5 s, a second glass plate (20 g) was placed on top of the cement drop, followed by a 100 g weight, totaling 120 ± 2 g. After 10 min, the weight was removed, and the largest and smallest diameters of the cement were measured using a digital caliper (0.001 mm). The difference between the largest and smallest diameters was not greater than 1.0 mm. All groups were tested in triplicate (*n* = 3).

### 2.3. Radiopacity

Radiopacity was assessed according to ISO 6876/2012 [25]. Fifteen specimens, 10.0 mm in diameter and 1.0 mm in height, were prepared using metal rings (*n* = 3). After preparation, the specimens were placed on occlusal radiographic films, each film receiving one specimen from each experimental group, along with a 1.0 mm control and an aluminum step wedge (scale 1–10 mm of Al). The films were X-ray sensitized using an X-ray machine operating at 70 kV, 10 mA, with exposure times of 0.3 s and a focus/film distance of 30 cm. Radiopacity quantification was performed through densitometric analysis using Adobe Photoshop CC 2017 software (Adobe Systems, San Jose, CA, USA), employing the formula proposed by Duarte et al. [26]: RD = [A × 2/B] + mm Al (immediately lower step), where ‘A’ is the difference between the radiographic density of the material (RDm) and that of the immediately lower aluminum step; ‘B’ is the difference between adjacent aluminum steps; and ‘2′ is the standard increment between the steps of the penetrometer. The results were expressed in millimeters of aluminum (mm Al).

### 2.4. Volumetric Change Analysis

**Figure 1:**
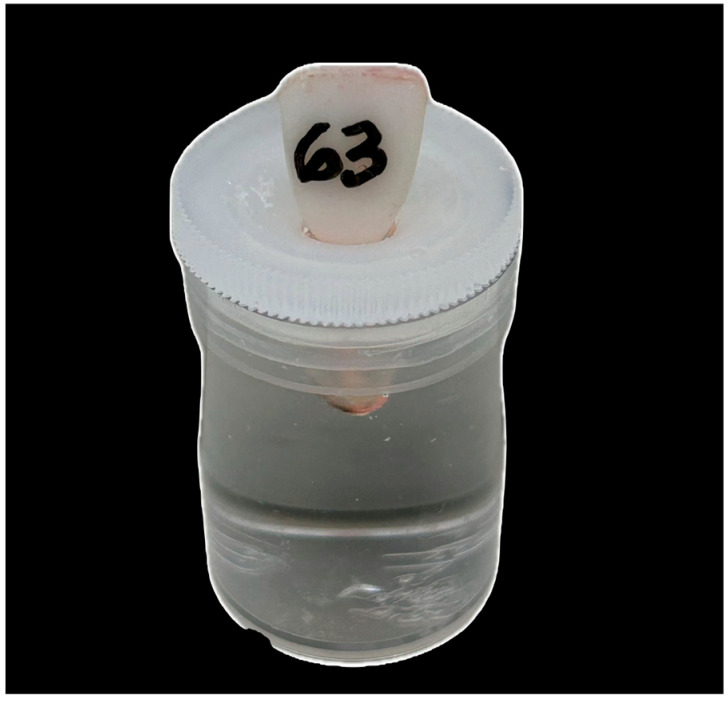
Experimental model showing a tooth specimen placed in an acrylic pot containing 10 mL of phosphate-buffered saline (PBS), with only the root portion immersed in the solution.

To analyze volumetric change, artificial teeth (IM do Brasil, São Paulo, Brazil) with 24 mm long circular canals were prepared using Genius Proflex rotary instruments (Medidenta, Las Vegas, NV, USA) up to a #40/0.04 file with a working length 1.0 mm short of the foraminal opening. Before obturation, the apical patency was confirmed using K-type #20 instruments (Dentsply-Maillefer, Konstanz, Germany). After biomechanical preparation, the teeth were obturated using the single-cone technique with size 40.04 taper cones (Tanari, Tanariman, Manacapuru, AM, Brazil). Immediately following obturation, the specimens were scanned using micro-CT and then placed in acrylic pots containing 10 mL of phosphate-buffered saline (PBS), with only the root portion in contact with the liquid—Figure 1. The specimens were maintained in an incubator at 37 °C and 95% humidity for 7 days. Pre- and post-scans were obtained using a micro-CT system (1174v2 SkyScan; Bruker-microCT, Kontich, Belgium) operating at 50 kV, 800 mA, with 360-degree rotation, and an isotropic resolution of 23.97 µm. The system includes a high-resolution camera (1304 × 1024 pixels). The images of each sample were reconstructed using specialized software (NRecon v.1.6.3; Bruker-microCT), generating transverse and axial slices of the samples’ structure. The surface segmentation and modeling were performed using the automatic CTAN v.1.12 software (Bruker-microCT) from the pre-scan images. For volumetric assessment of the material pre- and post-volume, the CTAN software (Bruker-microCT) was employed. The volume of material (mm^3^) was calculated for the apical 10 mm of each sample. Volumetric change was expressed as a percentage, calculated as the difference between the initial and final volumes. Negative values indicate volume expansion of the material [27].

### 2.5. Solubility

Solubility was tested according to ISO 6876:2012 [25]. The solubility test was performed using metal rings for the AH Plus Jet and gypsum rings for the calcium silicate-based sealers (P1, P2, P3 and AH Plus Bioceramic) with an internal diameter of 20 mm and a thickness of 1.5 mm, placed on a glass plate covered with cellophane paper and filled with the endodontic cement. A waterproof nylon thread was inserted into the material, followed by the placement of another glass plate, also covered with cellophane paper. The assembly was manually pressed to ensure that the plates made uniform contact with the mold. The samples were kept in an oven at 37 °C ± 1 °C and 95% ± 5% relative humidity for a period equivalent to three times the setting time of the tested material. After removing residues and loose particles, the samples were weighed on a precision analytical balance. They were then suspended by the nylon thread and placed in plastic containers containing 7.5 mL of distilled and deionized water, ensuring they did not touch the container walls and remained fully submerged. The samples were maintained under these conditions in an oven at 37 °C ± 1 °C and 95% ± 5% relative humidity for 7 days. After this period, they were removed from the containers, rinsed with distilled and deionized water, and excess water was removed with absorbent paper. Subsequently, they were kept in a desiccator for 24 h and weighed again. Solubility was determined by calculating the mass loss of each sample relative to its initial mass.

### 2.6. pH

The same acrylic teeth, prepared and obturated as described for the volumetric analysis, were used for pH determination. After obturation, the apices of the teeth were immediately immersed in acrylic pots containing 10 mL of phosphate-buffered saline and placed in an incubator at 37 °C and 95% humidity for the experiment. All pots were pre-treated with nitric acid to prevent any interference with the results. pH measurements were conducted at 3, 24, 72, and 168 h using a calibrated pH meter [2].

### 2.7. Statistical Analysis

The data were subjected to normality analysis using the Kolmogorov–Smirnov test, which indicated a normal distribution. For global comparison, ANOVA was applied, and individual comparisons were performed using the Tukey test. The statistical software used was GraphPad Prism 9.0 (GraphPad Software Inc., San Diego, CA, USA). A significance level of 5% was adopted.

## 3. Results

**Figure 2:**
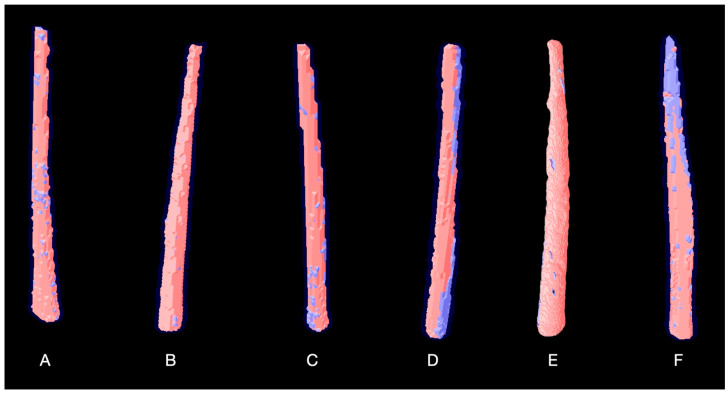
Representative micro-CT three-dimensional reconstructions of the evaluated root canal sealers: A—Proportion 1, B—Proportion 2, C—Proportion 3, D—Bio-C Sealer, E—AH Plus Jet, F—AH Plus Bioceramic.

Table 2 and Table 3 presents the mean and standard deviation for the initial and final setting times, flowability, radiopacity, solubility, and volumetric change. Statistically significant differences (*p* < 0.05) were observed in setting times among all groups, except for the initial setting time between P1 and AH Plus Bioceramic (*p* > 0.05). The radiopacity results showed that P1 and AH Plus Jet demonstrated the highest radiopacity values, with no statistically significant difference between them (*p* > 0.05). Bio-C Sealer presented the lowest radiopacity value (*p* < 0.05). Significant differences were found between P1 and both P3 and AH Plus Bioceramic, between P2 and AH Plus Bioceramic, and between AH Plus Jet and P2, P3, and AH Plus Bioceramic (*p* < 0.05). The calcium silicate-based sealer (P1, P2, P3, Bio-C Sealer and AH Plus Bioceramic) exhibited higher solubility than AH Plus Jet (*p* < 0.05). No statistically significant differences were observed in solubility among the three P (*p* > 0.05). Regarding volumetric change, Bio-C Sealer and AH Plus Bioceramic showed significantly higher volume alteration than P2 (*p* < 0.05). No significant differences were observed between P1, P3, AH Plus Jet, and the other materials (*p* > 0.05)—Figure 2.

Table 4 presents the mean and standard deviation of pH test results. In the intra-group evaluation, P1 and P2 exhibited an increase in pH between 24 and 72 h (*p* < 0.05). AH Plus Jet presented a significant pH increase between 3 and 24 h (*p* < 0.05), with no further significant changes in subsequent periods (*p* > 0.05). AH Plus Bioceramic showed significant pH increases between 3 and 168 h, 24 and 72 h, 72 and 168 h (*p* < 0.05). At 3 h, the pH of AH Plus Jet and Bioceramic was higher than that of P1, P2, and P3 (*p* < 0.05). From 24 h onwards, no significant differences were observed between groups (*p* > 0.05). At 72 h, the pH of P1, P2, P3, and AH Plus Bioceramic was higher than that of AH Plus Jet (*p* < 0.05). At 168 h, no differences were found between P1, P2, P3, and AH Plus Jet (*p* > 0.05), while AH Plus Bioceramic showed the highest pH (*p* < 0.05). Bio-C Sealer presented the highest pH values, with statistically significant differences between experimental time points (*p* < 0.05), except between 24 and 168 h (*p* > 0.05).

## 4. Discussion

Developing new endodontic sealers with optimized physicochemical and biological properties is crucial for endodontic treatment. Thus, the present study aimed to analyze three formulations with different calcium silicate/radiopacifiers proportions and compare them to the epoxy resin sealer AH Plus Jet and two ready-to-use commercial calcium silicate sealers, AH Plus Bioceramic and Bio-C Sealer.

The results of our study indicate that the lower calcium silicate content led to a faster setting, as well as higher flowability. P1 exhibited an initial setting time similar to AH Plus Bioceramic, although its final setting time was slightly longer. This suggests that AH Plus Bioceramic may contain components that accelerate setting more effectively. Notably, lithium carbonate, present in small concentrations in its composition, can modulate the setting reaction. This compound is commonly used in cement mixtures for tiling to accelerate the setting time [28,29]. AH Plus Bioceramic exhibited shorter setting times than AH Plus Jet, consistent with findings from a previous study [17].

All sealers evaluated exhibited radiopacity greater than 3 mm of Al. AH Plus Jet, which contains zirconium oxide and calcium tungstate in its composition as radiopacifiers, showed the highest value. A recent study [30] reported that the use of tantalum and zirconium oxide as radiopacifiers appears to be systemically safe. These findings support the clinical safety of these radiopacifiers in endodontic sealers, so they were selected for the different proportions (P1, P2 e P3). The results of our study are consistent with those found by Kandemir Demirci et al. [31], who reported similar radiopacity values for AH Plus Jet. The formulations P1, P2, and P3, which combined zirconium oxide with tantalum oxide, demonstrated greater radiopacity than AH Plus Bioceramic, with P1 achieving values comparable to those of AH Plus Jet. This is in line with the fact that using 30% zirconium oxide can yield values near 3 mm Al, as reported by Duarte et al. [26]. Increased radiopacity enhances the material’s contrast with mineralized structures such as dentin, cementum, and bone, facilitating its visibility in radiographs.

The lower proportions of calcium silicate and higher concentrations of radiopacifiers exhibited lower flowability. This could be due to the higher density of radiopacifiers, which might increase the viscosity of the material, making it less fluid. Additionally, the reduced proportion of calcium silicate may result in a thicker, less flowable mixture, potentially due to a decrease in the formation of hydrated compounds. These factors may collectively contribute to the observed reduction in flowability. The flowability of AH Plus Jet was similar to that observed in a previous study [23]. In our study, the AH Plus Bioceramic showed the highest flowability. While higher flowability may improve sealer penetration in complex anatomical areas and dentinal tubules, it could also lead to greater risk of extrusion, which can be associated with postoperative pain [32].

ISO standards set limits for the solubility of endodontic sealers to ensure their long-term effectiveness and safety. According to ISO 6876:2012, the maximum allowable solubility is 3% [25]. The results of the present study show solubility values within ISO standards only for the AH Plus Jet and P3. It is important to note that the solubility of hydraulic calcium silicate-based materials can be influenced by their water-to-powder ratio and the incorporation of radiopacifiers. For instance, the inclusion of tantalum oxide increases water demand, potentially leading to higher porosity and ion leaching, while reducing its content may improve solubility resistance by promoting a denser microstructure [33]. These findings highlight the need for careful adjustment of material composition to meet clinical requirements while maintaining physicochemical stability.

Interestingly, Bio-C Sealer, which exhibited the highest solubility and volumetric change among hydraulic sealers, also produced the highest pH values. This correlation may be explained by its greater ion release capacity, particularly calcium and hydroxyl ions, which is directly associated with the bioactivity of hydraulic cements [2,3,4,11,29]. While the solubility of calcium silicate-based sealers may be considered a disadvantage in terms of dimensional stability, it is precisely this characteristic that contributes to their reparative biological potential through enhanced bioactivity [1,12]. The solubility of tricalcium silicate sealers varies in the literature, possibly related to differences in protocols and methodologies used [1,11,29].

Ideally, endodontic sealers should exhibit minimal volumetric alteration after setting to prevent compromising the quality of the root canal filling [1,9,10]. The in vitro methods proposed by the ADA [34] and ISO [25] to assess solubility and dimensional stability do not fully replicate the clinical situation; therefore, our study employed a methodology previously reported in the literature, where only the radicular portion of the teeth remains in contact with the medium and is maintained in a controlled temperature and humidity environment [27]. Although natural teeth were not used in our study, a prior study demonstrated that artificial teeth can be considered a viable option for evaluating obturation quality using the single-cone technique with calcium silicate-based sealers [35]. To minimize the risk of bias, the single-cone technique was adopted, and all obturations were performed by a single experienced operator. Additionally, PBS solution was used as the storage medium, as previous research has shown that storage in PBS significantly reduces the volumetric change of calcium silicate-based sealers [36]. Our results indicate that the experimental sealers and AH Plus Jet exhibited lower volumetric changes compared to AH Plus Bioceramic and Bio-C Sealer. No studies have published data on the volumetric change in AH Plus Bioceramic; however, previous studies have reported similar volumetric changes for resin-based and calcium silicate-based sealers [20,36].

One of the main characteristics of calcium silicate-based sealers is the formation of calcium hydroxide during hydration, which leads to the alkalinization of the environment [4]. Although previous studies have shown that calcium silicate-based sealers can alkalinize the surrounding environment [2,4,27], our results revealed that none of the tested sealers could alkalinize the solution. This may be related to differences in the methodologies used to assess pH. The methodology employed in our study aimed to replicate clinical practice, where the sealers are placed within the root canal system, with no direct contact between the material and the extra-radicular environment.

The null hypotheses were rejected, as significant differences (*p* < 0.05) were observed in several physicochemical properties, including setting times, flowability, radiopacity, solubility, and volumetric change, between the P1, P2, P3, and the commercial sealers (AH Plus Jet and AH Plus Bioceramic). The different proportions exhibited less solubility and lower volumetric change compared to AH Plus Bioceramic, while radiopacity varied significantly among groups. Additionally, the different proportions of calcium silicate and radiopacifiers in the experimental sealers significantly influenced some properties, such as flowability and setting times, but not others, such as volumetric change among the experimental groups. The pH variations also showed significant differences at specific time points, further supporting the rejection of the null hypotheses. These findings indicate that the composition of sealers, particularly the proportions of calcium silicate and radiopacifiers, plays a critical role in determining their physicochemical properties.

Physical and chemical studies are crucial and should be conducted before the biological testing of endodontic sealers, as they provide essential data on the material’s properties. In our study, the P1 exhibited high radiopacity, high flow, low volumetric change, and a favorable setting time, while P3 was the only hydraulic sealer with solubility values within ISO [25] and ADA [34] limits. This suggests that the 50% calcium silicate/45% radiopacifier formulation may represent an optimal balance for clinical applications. The solubility of P1, P2, and AH Plus Bioceramic remained a drawback, indicating that compositional adjustments may be necessary for these formulations. Further biological and clinical studies are required to comprehensively evaluate these sealers, alongside additional tests such as bond strength (adhesion), push-out resistance, dentin-sealer interface analysis, and failure mode assessment.

## 5. Conclusions

Formulation P1 (30% calcium silicate/65% radiopacifier) demonstrated high radiopacity, favorable flowability, and low volumetric change, comparable to AH Plus Jet. Notably, formulation P3 (50% calcium silicate/45% radiopacifier) was the only hydraulic sealer with solubility values within ISO standards, whereas P1, P2, and the commercial calcium silicate-based sealers exhibited higher solubility than AH Plus Jet. Although compositional differences affected setting time and flowability, volumetric changes remained minimal for P1, P2, P3, and AH Plus Jet.

## Figures and Tables

**Table 1:** The manufacturer and composition of the tested sealers.

Sealers and Manufactures	Composition
Proportion 1 (P1—Bauru School of Dentistry, University of São Paulo, Bauru, Brazil)	Powder: 30% Calcium silicate, 2% Calcium chloride, 3% Calcium oxide, 40% Tantalum oxide, 25% Zirconium oxide. Liquid: 90% Propylene glycol, 5% Dimethyl sulfoxide, 5% Polycarboxylate
Proportion 2 (P2—Bauru School of Dentistry, University of São Paulo, Bauru, Brazil)	Powder: 40% Calcium silicate, 2% Calcium chloride, 3% Calcium oxide, 35% Tantalum oxide, 20% Zirconium oxide. Liquid: 90% Propylene glycol, 5% Dimethyl sulfoxide, 5% Polycarboxylate
Proportion 3 (P3—Bauru School of Dentistry, University of São Paulo, Bauru, Brazil)	Powder: 50% Calcium silicate, 2% Calcium chloride, 3% Calcium oxide, 30% Tantalum oxide, 15% Zirconium oxide Liquid: 90% Propylene glycol, 5% Dimethyl sulfoxide, 5% Polycarboxylate
AH Plus Bioceramic (Dentsply De Trey, Konstanz, Germany)	Zirconium dioxide (50–75%), Tricalcium silicate (5–15%), Dimethyl sulfoxide (10–30%), Lithium carbonate (<0.5%), Thickening agent (<6%)
AH Plus Jet (Dentsply De Trey, Konstanz, Germany)	Paste A: Bisphenol-A epoxy resin, Bisphenol-F epoxy resin, Calcium tungstate, Zirconium oxide, Silica, Iron oxide pigments. Paste B: Dibenzyldiamine, Aminoadamantane, Tricyclodecane-diamine, Calcium tungstate, Zirconium oxide, Silica, Silicone oil.
Bio-C Sealer (Angelus, Londrina, Brazil)	Calcium silicate, calcium aluminate, calcium oxide, zirconium oxide, iron oxide, silicon dioxide and dispersing agent

**Table 2:** Mean and standard deviation of initial and final setting time (in minutes) of the P1, P2, P3, AH Plus Jet, Bio-C Sealer, and AH Plus Bioceramic.

	Setting Time (min)	
Sealers	Initial	Final
P1	124.0 ± 1.0 ^a^	407 ± 1.0 ^a^
P2	155.0 ± 1.0 ^b^	426 ± 1.0 ^b^
P3	178.0 ± 1.0 ^c^	625 ± 1.0 ^c^
AH Plus Jet	847.3 ± 4.93 ^d^	1725 ± 1.0 ^d^
Bio C Sealer	237.7 ± 6.42 ^e^	446.7 ± 4.04 ^e^
AH Plus Bioc	115.0 +/− 3.0 ^a^	265 +/− 1.0 ^f^

Different superscript lowercase letters represent statistically significance differences (*p* < 0.05) in the same column.

**Table 3:** Mean and standard deviation of flowability (mm), radiopacity (mmAl), solubility (%), and volumetric change (%) of the P1, P2, P3, AH Plus Jet, Bio-C Sealer, and AH Plus Bioceramic.

Scheme	Flowability (mm)	Radiopacity (mm Al)	Volumetric Change (%)	Solubility (%)
P1	20.69 ± 0.01 ^a^	9.63 ± 1.39 ^ac^	2.07 ± 2.14 ª^b^	5.82 +/− 0.26 ^ab^
P2	19.70 ± 0.01 ^ab^	9.22 ± 1.25 ^a^	0.18 ± 3.10 ª	4.68 +/− 2.49 ^ab^
P3	19.19 ± 0.01 ^b^	6.93 ± 2.13 ^bd^	3.35 ± 5.22 ª^b^	2.68 +/− 1.57 ^a^
AH Plus Jet	25.47 ± 0.01 ^c^	11.73 ± 0.98 ^c^	1.65 ± 1.47 ª^b^	0.05 +/− 0.05 ^c^
Bio C Sealer	26.9 ± 0.99 ^d^	6.30 ± 0.20 ^d^	5.49 ± 4.24 ^b^	8.10 +/−2.15 ^b^
AH Plus Bioc	30.38 +/− 0.01 ^e^	6.58 +/− 0.59 ^b^	4.54 ± 2.02 ^b^	4.49 +/− 1.51 ^ab^

Different superscript lowercase letters represent statistically significance differences (*p* < 0.05) in the same column.

**Table 4:** Mean and standard deviation of pH of the P1, P2, P3, AH Plus Jet, Bio-C Sealer, and AH Plus Bioceramic.

pH				
Sealers	3 h	24 h	72 h	168 h
P1	6.86 ± 0.008 ^ABab^	6.86 ± 0.007 ^Aa^	6.88 ± 0.019 ^Bab^	6.87 ± 0.015 ^ABa^
P2	6.87 ± 0.011 ^ABabc^	6.87 ± 0.003 ^ACac^	6.88 ± 0.008 ^Bab^	6.88 ± 0.010 ^ABac^
P3	6.86 ± 0.005 ^Aa^	6.86 ± 0.006 ^ACac^	6.88 ± 0.015 ^Aa^	6.88 ± 0.043 ^Aac^
AH Plus Jet	6.88 ± 0.005 ^Aac^	6.87 ± 0.011 ^Aac^	6.86 ± 0.007 ^Ab^	6.87 ± 0.027 ^Aac^
Bio C Sealer	7.15 ± 0.012 ^Aa^	7.00 ± 0.011 ^Bbd^	7.25 ± 0.008 ^Cc^	7.04 ± 0.050 ^Bb^
AH Plus Bioceramic	6.88 ± 0.006 ^Ac^	6.85 ± 0.013 ^Ba^	6.88 ± 0.009 ^Aab^	6.91 ± 0.009 ^Cc^
Control	6.87 ± 0.013 ^Aac^	6.87 ± 0.013 ^Ac^	6.87 ± 0.013 ^Aab^	6.87 ± 0.013 ^Aac^

Different superscript lower-case letters represent statistically significance differences (*p* < 0.05) between periods studied in the same group. Different superscript upper-case letters represent statistically significance differences (*p* < 0.05) in the same column.

## Data Availability

The original contributions presented in this study are included in the article. Further inquiries can be directed to the corresponding author.
